# Views of healthcare professionals on recruiting to a psychosocial randomised controlled trial: a qualitative study

**DOI:** 10.1186/s12913-021-06817-2

**Published:** 2021-08-18

**Authors:** Trefor Aspden, Megan Armstrong, Marc Serfaty

**Affiliations:** 1 Division of Psychiatry, University College London, London, W1T 7NF UK; 2 Marie Curie Palliative Care Research Department, University College London, London, UK

**Keywords:** Qualitative, RCT, Randomised Controlled Trial, Psychosocial, Recruitment, Psychooncology

## Abstract

**Background:**

Randomised controlled trials, and in particular those of psychosocial interventions, often fail to recruit to target, resulting in underpowered trials with poor generalisability of findings. The objectives of this study were to explore the views of healthcare professionals on recruiting to psychosocial research studies, and to explore their views on factors that may hinder or facilitate recruitment.

**Methods:**

We conducted 14 semi-structured interviews, with healthcare professionals who had been involved in recruitment into a randomised controlled trial of a talking therapy for depression in patients with advanced cancer. Interviews were transcribed and analysed using thematic analysis.

**Results:**

Five primary themes were identified, comprised of 11 subthemes. Attitudes towards research were largely positive. Health care professionals identified lack of time and narrow screening criteria as barriers to recruitment, and also noted the tendency to withhold participants from research for reasons other than eligibility (e.g., gatekeeping). The engagement of the study team with the clinical recruitment site, and the frequent presence of a researcher in clinics, were noted as facilitating recruitment.

**Conclusions:**

Healthcare professionals involved in recruiting to trials of psychosocial interventions hold generally positive views of psychosocial research. However, they report that constraints including space and time limit their ability to recruit, and express anxieties about approaching patients for trial recruitment in the palliative phase of their illness. The findings from this study can inform how best to design trials, and in particular trials of psychosocial interventions, and train health care professionals for the study, to maximise recruitment.

## Background

Randomised controlled trials (RCTs) represent the gold standard for determining the clinical and cost effectiveness of health care interventions [1]. However, RCTs often fail to meet their recruitment targets: a review of trials funded by two UK bodies looked at 114 trials and found that less than a third reached their recruitment target, while half had to extend the recruitment period [2]. As an example of the problems that trials can face, one RCT of aromatherapy in advanced cancer found many patients were too ill to approach, a higher number than anticipated declined, and referrers acted as gate-keepers due to scepticism about treatment and the wish to reduce patient burden [3]. Failing to reach recruitment targets results in underpowered trials, with poor generalisability of findings [4].

Recruitment into trials in palliative care are beset by a unique combination of problems [4, 5], including those common to all types of trials such as care providers being too busy and “gatekeeping” by care providers, and those unique to palliative care such as patients’ disease burden. Gatekeeping refers to actions that control the provision of eligible patients [4]. Gatekeeping by care providers may be a problem in palliative care research because of the desire to protect patients, and a reluctance to ask them to spend their remaining time participating in research [6]. Data from an observational palliative care study suggests 24 % of palliative patients were excluded from research because of gatekeeping [7]. A survey of healthcare professionals views towards referring patients to palliative care trials suggests this desire to protect end of life patients is widespread [8]. However, this may be discordant with the wishes of palliative patients. A review of studies looking at the views of patients or carers towards participating in palliative care trials or research studies suggests palliative care patients are generally happy to be invited to participate in research, experience direct and indirect benefits (such as feelings of altruism) from participation, and are averse to others saying “no” on their behalf [9]. Similarly, patients with advanced cancer who had participated in symptom control trials generally regarded participating in the trial as a positive experience, irrespective of whether the trial had improved symptoms [10].

Trials of psychosocial interventions in cancer patients have also been noted to face unique recruitment issues. Firstly, cancer patients may be preoccupied with treating the physical disease, and therefore may not make use of psychosocial services [3, 11]. Secondly, participants that do want support may feel deterred by the prospect of randomisation to a non-preferred group [3]. Given the difficulties faced in recruitment to palliative care RCTs, it is important to establish what barriers hinder recruitment, and explore potential methods of enhancing recruitment.

In this paper, we present qualitative findings from our experience of recruitment to the CanTalk study, a RCT comparing cognitive behavioural therapy (CBT) to usual care for treating depression in people with advanced cancer [12]. Despite successfully recruiting 230 participants out of a target of 240, individual participating sites struggled with achieving recruitment targets. To meet recruitment targets expansion in number of sites (from an initial 4 hospices and oncology centres to 17) and extension of the recruitment period (from a 24 month recruitment period to 40 months) were required, and university employed researchers were deployed in research sites located close to the university to increase recruitment. Our objective in the present study was to explore the views of those involved in recruitment into the CanTalk RCT, what they perceive the barriers to recruitment to be, and what they think would facilitate increased recruitment.

## Methods

### Design

The study comprised qualitative interviews of healthcare professionals involved in recruiting to a RCT, the CanTalk study [12] (Controlled Trials ISRCTN07622709, registered 15 July 2011). The CanTalk RCT recruited participants with advanced cancer and depression from oncology centres and a hospice located in areas where referral to Improving Access to Psychological Therapy (IAPT) centres for depression was possible. It compared Cognitive Behavioural Therapy with Treatment as Usual for depression in these patients. Only patients living within the catchment areas for these IAPT centres were eligible to participate.

Ethical approval was provided by the London – Camberwell St Giles NRES committee, reference 11/LO/0376. The study was supported by the National Cancer Research Network clinical trials portfolio.

### Participants, recruitment and setting

Participants were healthcare professionals (*n* = 14), selected through purposive sampling to include a range of clinical and research focused occupations, to reflect the range of occupations involved in recruitment to the CanTalk trial. These participants were recruited through an email invitation, followed up by a phone call. Recruitment was conducted by researchers from University College London, employed on the CanTalk trial. Participants were recruited from the Christie NHS Foundation Trust, Barts Health NHS Trust, North Middlesex University Hospital, Weston General Hospital, Princess Royal University Hospital, South Tyneside NHS Foundation Trust, University College London Hospital, Whittington Health NHS Trust, and Marie Curie Hospice Hampstead. Participants’ involvement in the CanTalk RCT involved identifying relevant patients, approaching and discussing the study with patients, and screening for eligibility for the study.

### Topic guide

The experience of researchers employed on the CanTalk trial to recruit in oncology clinics suggested that some staff were reluctant to refer patients to the trial. With a view to exploring possible reasons for this, and attitudes of staff towards psychosocial research trials in general, a qualitative topic guide was developed [13], asking about (1) role in the oncology clinic; (2) previous research involvement; (3) views on trials other than clinical trials of investigational medicinal products (CTIMPs); (4) views about the CanTalk trial; (5) factors influencing recruitment; (6) feedback about the CanTalk trial; and (7) future research involvement. This topic guide is summarised in Table 1. The guide was structured to begin with broad open questions and progress to narrower more specific questions. We kept open the option of adapting or expanding the topic guide if new topics emerged during the interviews. However, as all topics arising appeared to fit well within the existing structure of the guide, no alterations were made.

**Table 1:** Summary of interview topic guide

1. Describe your role in the oncology clinics.
• Describe your current role? How did you come to be in this role? How do you feel about your role?
2. Have you been involved in any type of research in your role?
• Type of methodology? Duration? Personal research or study?
3. I would like to ask you a little bit now on your views on trials that are not of medicinal products.
• How often do you participate these? To what extent do you feel these are part of your remit?
4. I would like to ask you more specific questions now about your views on the CanTalk trial itself.
• What do you know about the CanTalk study? Views on the research being carried out by the university? Views on comparison of active treatment with usual care?
5. Now I will ask you questions about your participation in the CanTalk trial.
• How do your feelings about research influence involvement? How do your patient’s experiences of participation affect involvement? What factors may influence recruitment?
6. I would like to ask you now to tell me about any patient feedback you have had about CanTalk.
• Have any of your patients mentioned the trial? How do you feel the trial affected them?
7. Lastly I would like to ask you about your opinions on psychological research studies. Would you participate in this type of research in the future?
• Why do you say this? What might make you invest more in these types of trials?
8. Overall what did you think about the CanTalk study and do you have any suggestions for improvements to studies like these in the future?

### Interviews

Interviews took place between December 2015 and March 2016, after recruitment closure but within three months of recruitment ending. Informed consent was obtained prior to each interview. Interviews were conducted by researchers employed on the CanTalk study and trained in qualitative techniques, in a private room at the participants’ place of work, and lasted between 16 and 66 min. Interviews were recorded using a digital recording device. Interviews were conducted using a semi structured interview technique, based on the topic guide. This allowed for the interview to be directed by the topic guide, while also allowing for divergence from the guide to explore topics in greater depth or to explore issues raised by participants that were not specifically covered by the guide.

### Analysis

Recordings of interviews were transcribed verbatim. Data were analysed using thematic analysis based on commonly used guidelines [14], using Nvivo 11 software. We chose to use thematic analysis as it is not tied to any specific theoretical standpoint, and provides a flexible method that can be tailored to the research questions [14]. After familiarisation with the data, two researchers (TA and MA) independently coded the data and identified themes. These researchers then met to discuss the themes. Any differences were resolved through discussion. The analysis throughout, including the selection of text for coding and the search for themes, was guided by the study’s research questions. The analysis consisted of the following stages:

1. *Familiarisation with the data*. Researchers familiarise themselves with the data set.
2. *Generate codes*. Text considered relevant to the research questions was coded. Text could be coded once, or multiple times.
3. *Search for themes*. Codes were sorted into themes. Each theme comprised one or more codes of similar meaning.
4. *Review themes*. Themes were refined. First, codes within each theme were reviewed for coherence. Secondly, themes were reviewed to ascertain: validity of individual themes in relation to the data; a clear distinction between themes; and that the thematic map generated reflects the meaning in the data.
5. *Define and name themes*. For each theme, collated codes were read in order to derive a definition.
6. *Produce a narrative account*. A narrative account of the thematic map of the data was produced.

### Reflexive statement

The chief investigator [MS] and the trial manager [TA] of the CanTalk trial set up this qualitative study to understand the reasons for the difficulties with recruitment. It is therefore possible the researchers had preconceived ideas about why recruitment was challenging, and pre-existing biases and assumptions of researchers may have influenced the interview process. However, researchers had experience in exploring a topic in-depth through interviews, and every effort was made to develop an open and unbiased interview schedule. To ensure the analysis of the data was as unbiased as possible, another researcher [MA] not involved in the CanTalk study also independently analysed the data, and agreement was reached between the whole team about the identified themes.

## Results

Of 50 healthcare professionals invited, 14 agreed to participate. The most common reason for non-participation was a lack of response to the invitation to participate. All participants worked within either hospital oncology clinics or a hospice and all were involved in participant recruitment for the CanTalk study. Participants came from a range of sites, including those that struggled with recruitment for the study, and some that recruited well. Occupations and sex of participating healthcare professionals are provided in Table 2.

**Table 2:** Participant demographic data

Identifier	Sex	Role
P01	Female	Clinical Research Practitioner
P02	Female	Research Nurse
P03	Female	Clinical Nurse Specialist
P04	Male	Consultant Clinical Psychologist
P05	Female	Clinical Nurse Specialist
P06	Female	Staff Doctor
P07	Female	Clinical Trials Officer
P08	Male	Oncology Consultant
P09	Female	Research Nurse
P10	Female	Research Nurse
P11	Female	Palliative Care Consultant
P12	Female	Oncology Consultant
P13	Male	Research Nurse
P14	Female	Oncology Consultant

**Fig. 1:**
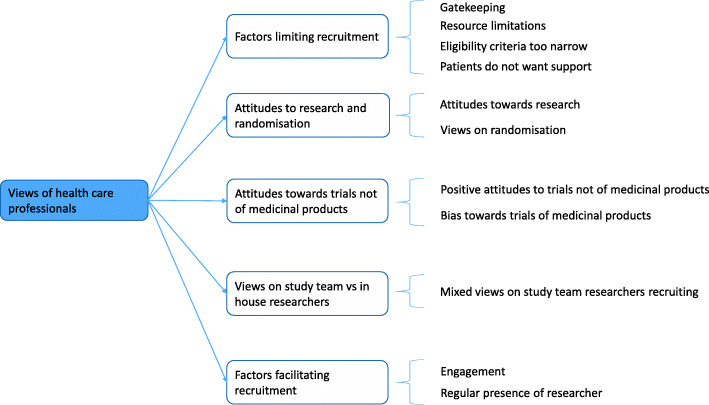
Thematic map of data

Five main themes emerged from the data: *(1) Factors limiting recruitment (2) Attitudes to research and randomisation; (3) Attitudes towards trials not of medicinal products; (4) Views on study team vs. in house researchers;* and *(5) Factors facilitating recruitment*. From these, 11 subthemes were identified. Saturation was reached in this analysis. A thematic map is provided in Fig. 1.

### Factors limiting recruitment

#### Gatekeeping

A number of healthcare professionals discussed pre-selecting patients. This included avoiding people *at the end of their life who are struggling* (P03), who were *in a hospital bed and looking pretty sick* (P03), and avoiding *people with really difficult personalities* (P14). Reasons suggested for gatekeeping included not wanting *to upset their patients* (P06) or to *give them any extra burden* (P10):

… *you’re not really going to be offering a therapy that they may not be around to benefit from …. and also put them to the inconvenience of going and sorting all this out* … (P03).

Some Healthcare professionals displayed an awareness of their tendencies to gatekeep:

*… if you could be a bit more objective about it and think ‘they meet the criteria I can go and speak to them’, I think maybe sometimes it, it stopped us from approaching people …* (P10).

One healthcare professional suggested that a researcher who is “*blind”* to the patients (i.e. who has no existing knowledge or preconceptions of the patient from previous contact with them) may be less likely to gatekeep:

… *sometimes coming in blind and not knowing all that information is fine because you can just discuss it with somebody and they can say yes or no*… (P03).

#### Resource limitations

Limitations in *time* (P02) and *space* (P10) were noted to limit recruitment. In terms of time, competing priorities (e.g. *always something more pressing to do*; P03) and demands of a *two hour wait in clinic* (P14) were noted. In terms of space, limited availability of *clinic rooms* (P10) was noted as a limiting factor:

… *they were welcoming for us to come in a see the patients um, but again its busy clinics its clinic time its clinic rooms, its finding that space* … (P10).

One individual expressed the view that *I don’t have dedicated time for research* (P11) and another noted the added burden of research on top of existing pressures:

*…they’re [the oncology clinic] understaffed at the moment so they feel it’s, research is like an added … it’s added work for them …* (P02).

#### Eligibility criteria too narrow

This theme encompassed the notion that the eligibility criteria were too narrow overall. *Broadening the patient eligibility* (P08) criteria or providing the trial intervention *on site* [P08] were noted as factors that would increase recruitment. This theme largely focused on the catchment area, with a number of healthcare professionals noting that the catchment area for treatment centres limited the number of eligible patients, restricted *what clinics we screened* (P09) and constituted a *postcode lottery* (P03).

… *we had a spreadsheet of nearly, I think it got to point where we had about eighty or ninety patients we had on that list, but then as these, like the barriers kept coming in we had to keep cutting out people …. cutting out people and the screening and then that got cut out, so the list just kept going smaller and smaller and smaller …* (P02).

#### Patients do not want or need support

This theme comprised the concept that many patients did not *need* (P01) or *want* (P08) support, including the rejection of formalised psychological support:

…*we had plenty of patients who both myself and the clinicians, like the consultants, really thought needed talking therapy because they would literally take up an hour of the medical oncologist’s time but when it came to offering the study they didn’t need any help* …. (P01)

Some patients were noted to *choose church over study* (P01). It was also noted that there may be less hopelessness among cancer patients and they *don’t feel as hopeless now as they maybe did five or ten years ago* (P07).

### Attitudes to research and randomisation

#### Attitudes towards research

Attitudes towards research were largely positive, for example commenting on the usefulness of research:

… *according to the results it might help many more in future so it’s like I’m being part of the future research evidence base that would be used probably five, ten years to come*… (P05).

Several participants commented that research helps to *build up the evidence base* (P12), *is the way forward* (P03), and that randomised controlled trials are *the gold standard* (P01). Other participants attitude towards research were more factual, noting that research and recruitment were *part of my job* (P05), or remit, for example:

… *certain groups of clinicians don’t want to get involved in research …, whereas … most oncologists I know wouldn’t feel like that cause they know that research is part of our work*… (P14).

#### Views on randomisation

Several healthcare professionals expressed that randomisation is necessary to answer the question, that it is *the only way that we can compare things* (P10), and provides a *scientifically robust test* (P08). However, along with this acknowledgement of the necessity and benefits of research there was also a view expressed that randomisation is a *short term sacrifice [to patients allocated to usual care] for long term gain* (P01). This suggests that while health care professionals understand the benefits of randomisation, it may be difficult for them to maintain a sense of equipoise:

*… though I’ve got patients at the moment who didn’t get the active arm, perhaps I, I say to myself ‘well in two years all of my patients will be getting this as standard treatment’ …* (P01).

Some healthcare professionals commented that randomisation to an intervention or usual care group was *no particular concern* (P03) because the patient could *still get help if they needed it* (P05):

*… it wasn’t like if they were, if they were randomised to that and they didn’t get any, the CBT it wasn’t that they couldn’t then have anything through the hospital …* (P10).

However, a number of healthcare professionals noted that it can be *difficult* (P13) or *hard knowing that … patients aren’t going to get what we really want them to get* (P01). Healthcare professionals also spoke of *worry* (P12) and feeling guilty about patients with depression being assigned to standard care:

… *it can be quite hard letting people know that they’ve not got the intervention and, if you know that people are you know really keen for it you can feel quite guilty* *…* (P10)

### Attitudes to trials other than Clinical Trials of Investigative Medicinal Products (non-CTIMP trials)

#### Positive attitudes to trials not of medicinal products

There was a commonly expressed theme that non-CTIMP trials are *very important* (P11, P12) and help meet patients’ holistic needs:

… *I think if were looking at trying to, uh improve patient care from a kind of a holistic perspectives … these kind of studies and other similar ones are vital*… (P08).

Some health care professionals also expressed the notion that both CTIMP and non-CTIMP trials hold equal importance, and recruitment centres *need a balance* (P13):

*… we need to do both, ‘cos we’re paid for both (yeah) and we need to give patients the chance for both …* (P02).

Several healthcare professionals noted advantages to non-CTIMP trials, such as being more *straightforward so you weren’t involving any drugs or treatment* (P03):

… *non-CTIMPs actually suit a hospital like the [name redacted] hospital very well, because they’re not so resource intensive*… (P01).

As well as noting that trials not of medicinal products could be more straightforward, healthcare professionals also noted that psychosocial research studies, which commonly involve completing outcome questionnaires with participants, can allow staff to spend more time with, *chat with* (P07), and better understand patients:

… I like the fact that the questionnaire studies in particular enable me to spend time, more time with the patients actually understanding them as people (mmm) so they are much more people than they are drug trial subject … (P01).

#### Bias towards trials of medicinal products

Several healthcare professionals reported a bias towards recruiting for trials of medicinal products. Consultants were noted to *be involved more with CTIMP trials* (P02), to be *just interested in medical oncology really* (P11), and to *favour their own studies* (P01),

*… you only had one haematologist who’d think of CanTalk but the other would think about all the other CTIMP trials and then, you say, oh what about CanTalk, they’re like, ‘um, no, no, no’ as in they push it to the back, that would be like the last resort …* (P02).

This bias was accompanied by a perception that the *clinical trials points system gives more … funding point to trusts for … patients that are recruited into drug trials* (P01):

*… they were probably told that we get more money for the CTIMP trials … so it’s all I suppose funding …, so originally that’s what it was, so that’s what they would concentrate on, ‘cos they need to bring more money to the Trust …* (P02).

It was also noted that non-CTIMP trials could *get pushed* [out] (P01), and that if there were treatment trials *patients will always get offered that first … as they might potentially improve their outcomes* (P10). Related to this preference for drug trials, there was a notion that time recruiting to non-drug trials could be frowned upon:

*… I was pushing it saying yes we should take part, this is something we can do cause at the time there were very limited breast studies … but yes the time was um, perhaps a little frowned upon…* (P03).

### Views on study team vs. in house researchers

#### Mixed views on advantages of UCL or in-house researchers recruiting for the study

Views on staff from the university trial team conducting recruitment, as opposed to staff employed by the recruitment site, were mixed. Advantages of in-house clinical staff recruiting included the *advantage from us being actually embedded within [the clinic]* (P13), having an established *relationship with a clinician* (P13), and *sometimes they [the patient] do it [participate] because they know you (P3)*:

*…they know us well enough to know that we are not going to offer them something that they don’t think might help…* (P14).

Several healthcare professionals expressed advantages to researchers employed directly on the trial recruiting, noting that when trial researchers became involved it *felt like an enormous weight off* (P03), that *we just wouldn’t of had the resource* (P04), and that UCL researchers were *more objective* (P10) in selecting patients. For example:

… *when your colleagues came from UCL the numbers went up again … because they looked, person, or this person is official and actually taken time to come from the University* … (P02).

### Factors facilitating recruitment

#### Engagement

It was deemed important for successful trial recruitment to maximise engagement with sites:

… *just really more engagement to stakeholders so that they are aware (yea) of what the study is what it involves* … (P12).

It was also deemed important to meet people face to face and involve all interested parties:

… *really meet somebody face to face and to explain the study personally, because on paper it might not very interesting and um then to involve all the people that may or may not have views on this matter*… (P03).

Expanding on the theme of engaging with all interested parties, it was suggested that presenting the research at *pan London meetings* (P03) of nurse specialists or organisations such as the *London Cancer Alliance* (P04) would be useful. Presenting at meetings of nurse specialists and similar meetings will help ensure that those actively involved in recruitment are aware of the trial and have the opportunity to provide their feedback.

Engaging regularly was deemed important, for example through *regular site visits* (P01).

… *I think just engagement centrally umm you know keeping the clinicians who are involved in the trial up to date means not just it doesn’t just mean having a newsletter* … (P12).

This theme can be summed up as the importance of maximising engagement, including face to face engagement and engagement with all interested parties, and maintaining regular engagement with recruitment sites.

#### Regular presence of researcher

The regular presence of a researcher in clinics and in multi-disciplinary team meetings was noted as a key factor in facilitating recruitment:

…*I find our MDT’s every week a really useful time for people to make their presence remembered even if it can’t be every-, it doesn’t have to be every week it’s just as long as it’s often enough (yeah), s-, so that we keep a, thinking it through, remembering that we need to ask patients*… (P06).

It was deemed useful to have *somebody there to catch all the patients that come into those clinics* (P03), and to have a trial researcher who *forces themselves on the site* (P01):

… *that’s absolutely the key to recruitment, unless people jog us as clinicians regularly …. or sit and embed themselves in our meetings …. we are not going to be avid recruiters because there’s so much else to think about*… (P11).

## Discussion

Given the problems recruiting into RCTs, particularly within palliative care and trials of psychological interventions, it is important to explore why trials may under-recruit. The present study conducted qualitative interviews with health care professionals who had been involved in recruitment for a trial of a psychosocial intervention in an advanced cancer population. Overall, the study identified 5 main themes and 11 subthemes. Our findings suggest that whilst health care professionals felt that research was important, they did not have the time to engage in recruitment, and that trials of medicinal products were often prioritised over trials of psychosocial interventions.

Gatekeeping was identified in the present study as one of the barriers to recruitment, consistent with previous research which has identified a widespread desire to protect palliative patients from research [8]. Reasons for withholding patients from research have been described in previous research into the views of healthcare professionals involved in recruitment to trials [15]: doctors described being uncomfortable recruiting particular groups or individual patients, and nurses can make ‘clinical’ decisions about whom to approach based on personal viewpoints and avoid bothering certain patients [15].

Reasons that healthcare professionals in the present study gave for withholding patients included not referring patients who were too sick, avoiding patients with difficult personalities, and not wanting to increase patient burden. Not wanting to increase the burden on palliative patients has emerged as a reason for gatekeeping in several previous studies [6, 8, 16]. The consequence is that these patients are denied the opportunity to participate. Indeed, a review of palliative care patients attitudes suggests they are generally happy to be asked to participate, that they experienced both direct and indirect (such as feelings of altruism and meaning gained from helping to improve services for others in the future) benefits from participation, and that they are adverse to having other people say “no” on their behalf [9]. Thus, what is viewed by health care professionals as protecting a patient may be viewed by the patient very differently, as denying them the opportunity to make their own autonomous decisions.

Among factors influencing recruitment a primary theme was *resource limitations*, including time and space constraints and the competitive nature of recruitment, in which a number of trials typically compete for the limited time of staff. This theme is consistent with previous studies in which healthcare professionals stated that time is a factor limiting recruitment (e.g. [17–19]). As site resources are finite, one way to facilitate recruitment is for university researchers to recruit in clinics. This is noted to increase recruitment in the *Views on study team vs. in house researchers* theme, and our experience in running the CanTalk trial suggested that deploying university researchers to recruit in oncology clinics was essential in meeting the trials recruitment targets.

Narrow eligibility criteria was perceived as a key barrier, and has previously been noted as one of the main reasons for under recruitment into RCTs [20–22]. Indeed, researchers sometimes have to broaden the eligibility criteria in order to reach recruitment targets (e.g., [3]). Furthermore, healthcare professionals stated that those who are eligible often reject psychological support. This finding is consistent with previous research showing that patients declining to participate in trials of psychological interventions often feel they do not require a psychological intervention [3].

Attitudes towards research in general were mainly positive, and healthcare professionals stated that psychosocial trials are worthwhile. However it was noted that research was extra work, and not part of their remit. Additionally, there was the notion that consultants prioritise trials of medicinal products. This bias towards trials of medicinal products, which are perceived to “count more” and provide more reward to the recruitment sites, may partly explain why trials of psychosocial interventions can be particularly problematic to recruit to [23]. Although the UK Clinical Research Network does not prioritise drug trials, the present research suggests that consultants’ preference for trials which may affect physical outcomes may encourage recruitment efforts to be focused on drug trials.

The theme *Engagement* is consistent with research demonstrating that sites receiving regular updates on the trial is useful [22]. Several trials have documented attempts to maximise engagement (e.g. [24]). Healthcare professionals in the present study also noted that the regular presence of the researcher in clinic and meetings maximised recruitment, which was consistent with our observation that recruitment increased at sites when university researchers were deployed to them.

While the paper looked primarily at themes across the whole group of participants, some differences between the main subgroups of participant occupation can also be observed. As can be expected given the nature of their roles, participants whose careers were focused on research (research nurse/practitioner/coordinator) spoke in greater detail and more frequently about the importance of the randomisation process in providing an evidence base. In contrast to those with a research focused role, clinical practitioners (clinical nurses/consultants) noted not having dedicated time for research, and also time spent on research being frowned upon. This can be an important consideration during trial set-up, as agreements may sometimes be made for participant identification or screening to be conducted by clinical staff, who may not subsequently have the time for these activities in a busy clinical setting.

### Recommendations

Based on the present research, a number of recommendations can be made which should be considered during the design, set-up and running of clinical trials of psychosocial interventions, particularly those conducted within advanced cancer populations.

Firstly, during trial design, formulating a realistic estimation of the time that identification, screening, and referral of patients will take, and establishing whether healthcare professionals in referral sites will have dedicated time for these tasks, may help to ameliorate potential recruitment problems. This is particularly important given the competitive nature of recruitment, with multiple studies competing for staff time, and especially given that psychosocial research may be “pushed out” in favour of trials of medicinal products. Ring-fencing staff time dedicated to the study is required.

Secondly, educating staff involved in trial recruitment about the importance of RCTs may be beneficial to reducing gatekeeping. This could be done initially in set-up meetings. Providing information on the phenomena of gatekeeping, how the wish to protect patients is common among healthcare professionals [8] while being inconsistent with the views of palliative care patients [7], and possibly providing some quotes from the research literature on the benefits that palliative care patients experience from participating in research, may be beneficial in reducing gatekeeping.

Thirdly, as recruitment is notoriously difficult within palliative care trials and trials of psychosocial interventions, it will be important during trial design to make the eligibility criteria as broad as possible. We suggest that it is good practice to carefully review each criteria to see if there is any way to broaden eligibility.

### Study limitations

We approached 50 potential participants, out of whom 14 (28 %) agreed to participate. As this is a relatively low uptake, there may be selection bias, and individuals who participated may have more positive attitudes towards psychosocial research. The interviewers were researchers employed directly on the CanTalk trial, and in some instances would have had previous interactions with the interviewees during the course of recruitment. While this may potentially have influenced responses, we have no evidence to indicate that it did, and health care workers seemed to open up freely about their own biases and tendencies, such as gatekeeping. We did not seek the views of patients in the present study, which would provide valuable viewpoints on the topics explored. The topic guide used in this study did not explore what healthcare professionals said to patients about the study, which may have influenced recruitment into the CanTalk RCT. Actively testing these recommendations, for example through the use of embedded trials [25], is needed before we can be sure of the efficacy of any of these recommendations.

Notwithstanding these limitations, this study is the first to have investigated the views of health care professionals recruiting to a trial of a psychosocial intervention in an advanced cancer population. It has broadened our knowledge on factors facilitating and hindering recruitment into trials of psychosocial interventions in palliative care, and on the views held by the healthcare professionals tasked with recruitment into these trials. The findings will hopefully facilitate recruitment into RCTs, and reduce the chance of these requiring extensions in time and costs.

## Conclusions

The views of those recruiting to randomised controlled trials provide insight into what may block or facilitate recruitment. Our findings suggest that whilst health care professionals felt that research was important, they did not have the time to engage in recruitment. Healthcare professionals also describe withholding participants from research (gatekeeping). Realistically estimating the time involved in recruitment and protecting or “ring-fencing” recruitment time, educating recruitment staff about the importance of RCTs, and keeping inclusion criteria as broad as possible are recommended. These findings can help inform those setting up randomised controlled trials to minimise potential hurdles.

## Data Availability

The datasets used and/or analysed during the current study are available from the corresponding author on reasonable request.

## Abbreviations

CBT: Cognitive Behavioural Therapy
RCT: Randomised controlled trial

## References

[1] Odgaard-Jensen J, et al., Randomisation to protect against selection bias in healthcare trials. Cochrane Database Syst Rev 2011(4). 10.1002/14651858.MR000012.pub3 PMC7150228 21491415

[2] McDonald AM (2006). What influences recruitment to randomised controlled trials? A review of trials funded by two UK funding agencies. Trials.

[3] Westcombe A, et al. Learning the hard way! Setting up an RCT of aromatherapy massage for patients with advanced cancer. Palliat Med. 2003;17(4):300–7. 10.1191/0269216303pm769rr 12822844

[4] Kirchhoff KT, Kehl KA (2008). Recruiting participants in end-of-life research. Am J Hospice Palliat Med.

[5] O’Mara AM (2009). Challenges to and lessons learned from conducting palliative care research. J Pain Symptom Manag.

[6] Kars MC (2016). A systematic review of reasons for gatekeeping in palliative care research. Palliat Med.

[7] Stone PC, et al. Factors affecting recruitment to an observational multicentre palliative care study. BMJ Support Palliat Care. 2013;3(3):318-23. 10.1136/bmjspcare-2012-000396 PMC3756458 24644750

[8] White C, Gilshenan K, Hardy J (2008). A survey of the views of palliative care healthcare professionals towards referring cancer patients to participate in randomized controlled trials in palliative care. Support Care Cancer.

[9] White C, Hardy J (2010). What do palliative care patients and their relatives think about research in palliative care?—a systematic review. Support Care Cancer.

[10] Middlemiss T (2015). Symptom Control Trials in Patients With Advanced Cancer: A Qualitative Study. J Pain Symptom Manage.

[11] Michalec B (2005). Exploring the multidimensional benefits of breast cancer support groups. J Psychosoc Oncol.

[12] Serfaty M (2016). The clinical and cost effectiveness of cognitive behavioural therapy plus treatment as usual for the treatment of depression in advanced cancer (CanTalk): study protocol for a randomised controlled trial. Trials.

[13] Serfaty M (2019). Manualised cognitive behavioural therapy in treating depression in advanced cancer: The CanTalk RCT. Health Technol Assess.

[14] Braun V, Clarke V (2006). Using thematic analysis in psychology. Qual Res Psychol.

[15] Donovan JL (2014). Clear obstacles and hidden challenges: understanding recruiter perspectives in six pragmatic randomised controlled trials. Trials.

[16] Casarett DJ, Karlawish J, Hirschman KB (2002). Are hospices ready to participate in palliative care research? Results of a national survey. J Palliat Med.

[17] Bill-Axelson A (2008). Experiences of randomization: interviews with patients and clinicians in the SPCG-IV trial. Scand J Urol Nephrol.

[18] French C, Stavropoulou C (2016). Specialist nurses’ perceptions of inviting patients to participate in clinical research studies: a qualitative descriptive study of barriers and facilitators. BMC Med Res Methodol.

[19] Mason V (2007). GPs’ experiences of primary care mental health research: a qualitative study of the barriers to recruitment. Fam Pract.

[20] Gotay CC (1991). Accrual to cancer clinical trials: directions from the research literature. Soc Sci Med.

[21] Paramasivan S (2011). Key issues in recruitment to randomised controlled trials with very different interventions: a qualitative investigation of recruitment to the SPARE trial (CRUK/07/011). Trials.

[22] Elliott D, et al. Understanding and improving recruitment to randomised controlled trials: qualitative research approaches. Eur Urol. 2017;72;789-98. 10.1016/j.eururo.2017.04.036 28578829

[23] Moyer A (2009). Lessons to be learned from 25 years of research investigating psychosocial interventions for cancer patients. Cancer J.

[24] Jordhøy MS (1999). Challenges in palliative care research; recruitment, attrition and compliance: experience from a randomized controlled trial. Palliat Med.

[25] Madurasinghe VW (2016). Guidelines for reporting embedded recruitment trials. Trials.

